# Sulphur in Sugar

**Published:** 1890-09

**Authors:** 


### SULPHUR IN SUGAR.

We hear that a great deal of sulphur is used in the manufacture of sugar, and in no country in the world is it employed to a greater extent than in Louisiana. Sulphur is applied to cane juice in the form of gas, and it makes the product, both of sugar and molasses, lighter and brighter in appearance, planters claiming that it enhances the value from three to five cents on molasses, and that sugar has a brighter color and requires less washing to produce the same tone. The method generally adopted is to burn sulphur in a small brick oven. The fumes of the sulphur are carried by a pipe into a barrel of water, and the sulphurous gas coming into contract with the water is cleansed from sulphuric acid. The fumes thus purified pass from the barrel by means of a pipe into sulphuring chambers, which is constructed of wood in such a manner that the juice is constantly coming in and going out, and an arrangement is made so that the juice will fall in the form of rain or spray, the effect being to bleach out the coloring matter contained in the juice. Some manufacturers claim that a great deal of the sugar is destroyed by coming in contract with this sulphurous gas which contains a considerable quantity of sulphuric acid, and that by a little carelessness in applying this acid to the cane juice thousands of dollars a year have been lost in the larger manufactories. The question has been raised and discussed largely by scientists and pure-food men as to whether the sulphur affected the sugar so as to make it injurious to health, some claiming that it does, and some claiming that it does not. Where so many doctors disagree, it is extremely difficult to determine whether bleached sugar is harmful or not. The existence of sulphurous acid in molasses is what causes it so often to corrode metal vessels of various kinds with which it is brought in contact. It may be taken for granted that any substance that would corrode an iron pan or a copper vessel is hardly fit for human consumption.

